# Supplementary material for: Meta-Analysis and Gene Set Enrichment Relative to ER Status Reveal Elevated Activity of MYC and E2F in the “Basal” Breast Cancer Subgroup
Source: PLoS One. 2009 Mar 9;4(3):e4710. doi: 10.1371/journal.pone.0004710 (PMC2650420; doi:10.1371/journal.pone.0004710)
Supplement: Dataset S1 — GSEA results from first screen. The file contains a zipped archive of the HTML results of the second GSEA screen. Reports for enriched or depleted datasets may be accessed from the “index.html” link within the my_analysis.GseaPreranked.1219975950625.rpt.zip ZIP archive. Enrichment plots are available for each of the 50 most enriched and depleted gene sets. (13.32 MB ZIP) [file pone.0004710.s001.zip › my_analysis.GseaPreranked.1219975950625.rpt.zip!//FRASOR_ER_DN.html]

Details for gene set FRASOR\_ER\_DN[GSEA]

|  || Dataset | zSco.ERneg.to.ERpos\_collapsed |
| Phenotype | NoPhenotypeAvailable |
| Upregulated in class | na\_neg |
| GeneSet | FRASOR\_ER\_DN |
| Enrichment Score (ES) | -0.45340952 |
| Normalized Enrichment Score (NES) | -1.9238529 |
| Nominal p-value | 0.0 |
| FDR q-value | 0.01440259 |
| FWER p-Value | 0.367 |
Table: GSEA Results Summary

  

Fig 1: Enrichment plot: FRASOR\_ER\_DN      
 Profile of the Running ES Score & Positions of GeneSet Members on the Rank Ordered List

  

| PROBE | GENE SYMBOL | GENE\_TITLE | RANK IN GENE LIST | RANK METRIC SCORE | RUNNING ES | CORE ENRICHMENT || 1 | FOLH1 |  |  | 343 | 4.822 | 0.0104 | No |
| 2 | TM4SF1 |  |  | 639 | 4.054 | 0.0186 | No |
| 3 | ANXA3 |  |  | 1142 | 3.317 | 0.0056 | No |
| 4 | CD24 |  |  | 1159 | 3.286 | 0.0291 | No |
| 5 | KRT7 |  |  | 1262 | 3.180 | 0.0453 | No |
| 6 | ASB9 |  |  | 1472 | 2.973 | 0.0519 | No |
| 7 | KIAA0922 |  |  | 1784 | 2.705 | 0.0487 | No |
| 8 | TGFB2 |  |  | 2217 | 2.375 | 0.0340 | No |
| 9 | L1CAM |  |  | 2343 | 2.291 | 0.0418 | No |
| 10 | DBN1 |  |  | 2438 | 2.229 | 0.0514 | No |
| 11 | NDRG1 |  |  | 2463 | 2.218 | 0.0663 | No |
| 12 | KYNU |  |  | 3045 | 1.911 | 0.0368 | No |
| 13 | CTSL |  |  | 3453 | 1.729 | 0.0191 | No |
| 14 | IFI30 |  |  | 3555 | 1.684 | 0.0241 | No |
| 15 | LITAF |  |  | 3643 | 1.642 | 0.0299 | No |
| 16 | PRPS1 |  |  | 3889 | 1.556 | 0.0231 | No |
| 17 | MYO1B |  |  | 4100 | 1.473 | 0.0183 | No |
| 18 | ASPH |  |  | 6222 | 0.741 | -0.1360 | No |
| 19 | GNE |  |  | 6402 | 0.682 | -0.1444 | No |
| 20 | NFRKB |  |  | 6770 | 0.563 | -0.1679 | No |
| 21 | MATN2 |  |  | 7154 | 0.435 | -0.1935 | No |
| 22 | IL1R1 |  |  | 7459 | 0.325 | -0.2140 | No |
| 23 | CBX6 |  |  | 7571 | 0.281 | -0.2202 | No |
| 24 | EPOR |  |  | 7600 | 0.269 | -0.2203 | No |
| 25 | LHFPL2 |  |  | 7616 | 0.263 | -0.2195 | No |
| 26 | EFNA1 |  |  | 8130 | 0.063 | -0.2577 | No |
| 27 | CTSH |  |  | 8271 | 0.008 | -0.2682 | No |
| 28 | MMD |  |  | 8749 | -0.188 | -0.3028 | No |
| 29 | IRF6 |  |  | 8908 | -0.263 | -0.3127 | No |
| 30 | LAMB1 |  |  | 9080 | -0.350 | -0.3230 | No |
| 31 | CLU |  |  | 9333 | -0.475 | -0.3384 | No |
| 32 | ENC1 |  |  | 9363 | -0.491 | -0.3369 | No |
| 33 | ERBB2 |  |  | 9498 | -0.560 | -0.3428 | No |
| 34 | MXD4 |  |  | 9642 | -0.639 | -0.3488 | No |
| 35 | INHBB |  |  | 9653 | -0.647 | -0.3447 | No |
| 36 | GNS |  |  | 10327 | -1.046 | -0.3876 | No |
| 37 | ALDH4A1 |  |  | 10425 | -1.102 | -0.3866 | No |
| 38 | ARNT2 |  |  | 11302 | -1.826 | -0.4390 | Yes |
| 39 | STXBP1 |  |  | 11435 | -1.972 | -0.4341 | Yes |
| 40 | ECH1 |  |  | 11692 | -2.212 | -0.4368 | Yes |
| 41 | ABCG1 |  |  | 11792 | -2.319 | -0.4268 | Yes |
| 42 | STC1 |  |  | 11958 | -2.513 | -0.4204 | Yes |
| 43 | SLIT2 |  |  | 12017 | -2.581 | -0.4054 | Yes |
| 44 | BLNK |  |  | 12155 | -2.786 | -0.3948 | Yes |
| 45 | PPP1R3C |  |  | 12191 | -2.833 | -0.3762 | Yes |
| 46 | PVALB |  |  | 12208 | -2.849 | -0.3560 | Yes |
| 47 | IER3 |  |  | 12423 | -3.140 | -0.3485 | Yes |
| 48 | FMO5 |  |  | 12445 | -3.172 | -0.3263 | Yes |
| 49 | DUSP1 |  |  | 12483 | -3.240 | -0.3047 | Yes |
| 50 | MB |  |  | 12574 | -3.374 | -0.2862 | Yes |
| 51 | BIK |  |  | 12575 | -3.376 | -0.2608 | Yes |
| 52 | NELL2 |  |  | 12593 | -3.420 | -0.2364 | Yes |
| 53 | BCAS1 |  |  | 12594 | -3.421 | -0.2107 | Yes |
| 54 | ABCC5 |  |  | 12662 | -3.546 | -0.1891 | Yes |
| 55 | TGFB3 |  |  | 12734 | -3.702 | -0.1667 | Yes |
| 56 | BMP4 |  |  | 12783 | -3.824 | -0.1416 | Yes |
| 57 | LAMB2 |  |  | 12959 | -4.349 | -0.1221 | Yes |
| 58 | DUSP4 |  |  | 13001 | -4.456 | -0.0917 | Yes |
| 59 | HIST2H2BE |  |  | 13073 | -4.726 | -0.0616 | Yes |
| 60 | PIB5PA |  |  | 13125 | -4.921 | -0.0285 | Yes |
| 61 | SELENBP1 |  |  | 13238 | -5.739 | 0.0062 | Yes |
Table: GSEA details [plain text format]

  

Fig 2: FRASOR\_ER\_DN: Random ES distribution      
 Gene set null distribution of ES for **FRASOR\_ER\_DN**
